# Systemic mastocytosis mimicking blastic plasmacytoid dendritic cell neoplasm: a case report

**DOI:** 10.1186/s13000-023-01301-3

**Published:** 2023-02-10

**Authors:** Xin Zhang, Jing Han, Na Zhu, Yuan Ji, Yingyong Hou

**Affiliations:** Department of Pathology, Fudan University Zhongshan Hospital, 180 Fenglin Road, Shanghai, 200032 China

**Keywords:** Systemic mastocytosis, KIT, Blastic plasmacytoid dendritic cell neoplasm, Avapritinib, Case report

## Abstract

**Background:**

Systemic mastocytosis (SM), a rare myeloid neoplasm, is defined as a clonal and neoplastic proliferation of mast cells in at least one extracutaneous organ(s). The pathologic diagnosis and treatment of SM are challenging.

**Case presentation:**

We presented a 44-year-old male patient who had endured abdomen discomfort for 4 years and diarrhea for 5 months. Colonoscopy and PET/CT found a protuberant lesion in the cecum with adjacent lymphadenopathy. Histopathology of the cecum biopsy showed diffuse infiltration of medium-sized round/oval cells in lamina propria with immunohistochemical expressions of CD45, CD117, CD25, CD68, CD123, CD56, CD4, and CD35, mimicking blastic plasmacytoid dendritic cell neoplasm. Sanger sequencing revealed missense mutation (D816V) in the exon 17 of KIT gene. Serum tryptase level was 38.56 ng/ml. No abnormality was found in skin examination and bone marrow biopsy. No primitive cells were observed in bone marrow smear and peripheral blood smear. The diagnosis of aggressive SM with intestinal tract involvement was established. The patient received avapritinib treatment at an initial dosage of 200 mg once daily and exhibited dramatic clinical improvement but memory impairment within 1 month. No recurrence was observed in 1-year follow-up at the adjusted avapritinib dose (75 mg once daily).

**Conclusions:**

SM is very rare and should be considered in patients with long-term diarrhea symptoms and hematopoietic/lymphoid-appearing tumors. KIT D816V mutation contributes to the differentiation of CD123, CD4, and CD56 immunoreactive SM from blastic plasmacytoid dendritic cell neoplasm. The rare side-effect of memory impairment in this case helps to accumulate the experience of avapritinib in treating KIT D816V-mutant SM.

## Background

Mastocytosis, a unique and orphan disease, refers to a clonal and neoplastic proliferation of mast cells in one or more organ(s) [1]. The clinical manifestation of mastocytosis is heterogeneous, ranging from skin lesions that can spontaneously regress to highly aggressive neoplasms with multiple organ failure and poor survival [2]. According to the 2017 revised World Health Organization (WHO) classification of tumors of hematopoietic and lymphoid tissues, mastocytosis is broadly divided into three categories: cutaneous mastocytosis, systemic mastocytosis (SM), and mast cell sarcoma [1]. SM is characterized by cohesive aggregation of abnormal mast cells in at least one extracutaneous organ(s), with or without skin damage.

The diagnosis of intestinal SM is challenging due to its rarity and nonspecific symptoms, requiring integration of clinical, laboratory, morphological, immunohistochemical, and molecular genetic data. SM with immunoreactivity for key markers of blastic plasmacytoid dendritic cell neoplasm (BPDCN), including CD123, CD4, and CD56 has rarely been documented. SM is considered incurable, while recent studies have revealed encouraging responses following treatment with tyrosine kinase inhibitors, such as imatinib and the 2nd generation agent avapritinib [3, 4]. Particularly, avapritinib, as a selective inhibitor against KIT D816V, has exhibited dramatic efficiency in most SM patients resistant to imatinib. Here, we report a rare case of KIT D816V mutant SM which has similar phenotype with BPDCN. By being treated with avapritinib, this patient recovers well but shows a rare side effect of memory impairment.

## Case presentation

A 44-year-old man presented to his primary physician with complaints of right lower abdomen discomfort for 4 years and diarrhea for 5 months. The symptoms were aggravated within 1 week.

**Fig. 1:**
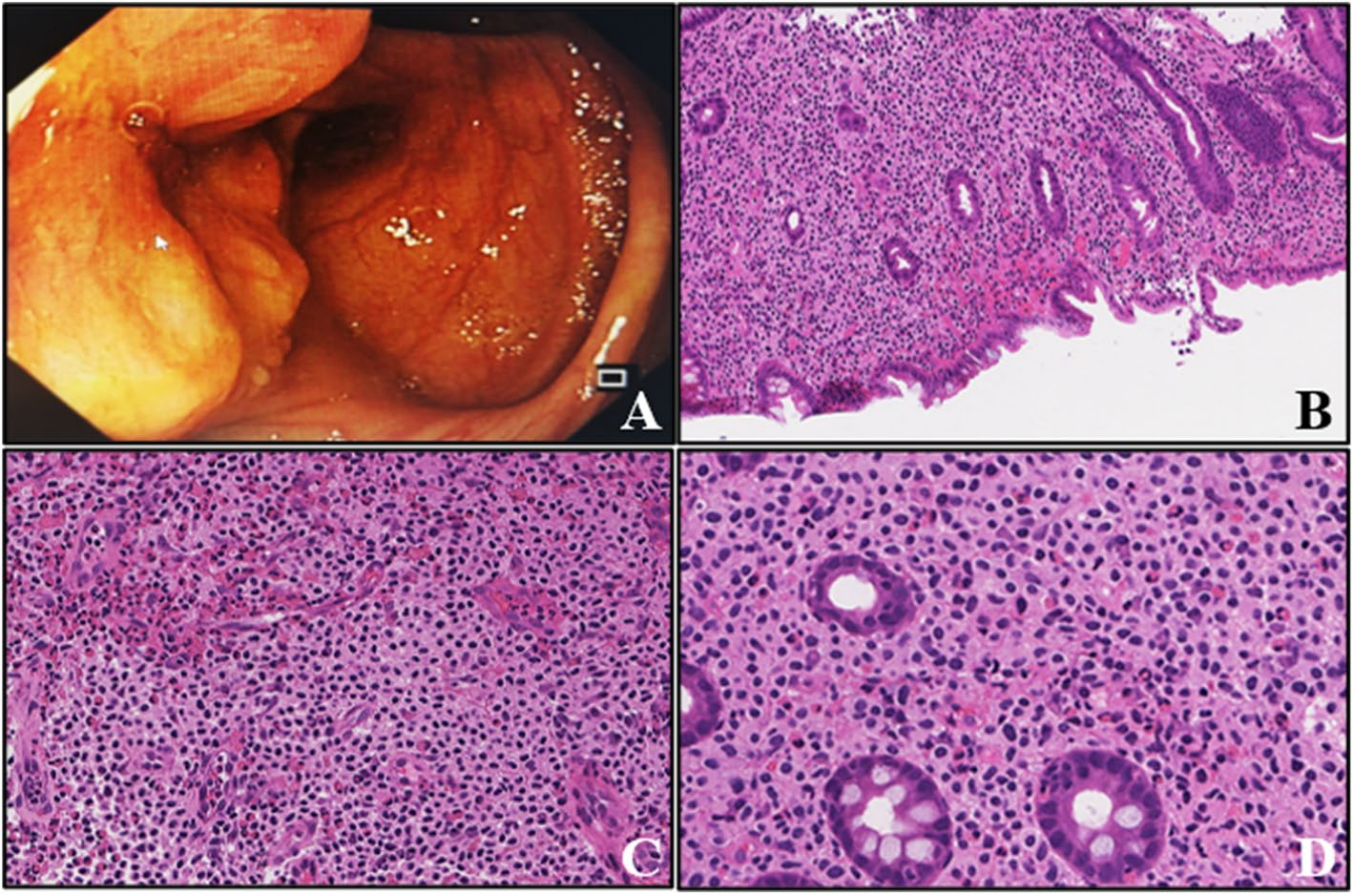
Endoscopic and morphological findings of the patient with systemic mastocytosis. **A** Colonoscopy showing a 3.0 cm × 3.0 cm protuberant lesion in the cecum with erosion, necrosis, and ulcer formation on the surface. **B** Biopsy specimen of the intestinal lesion depicted in (**A**), showing expanded lamina propria by medium-sized mast cells (hematoxylin and eosin stain, × 40 magnification). **C** Round/oval mast cells with a mixture of eosinophil cells (hematoxylin and eosin stain, × 100 magnification). **D** Atypical mast cells with abundant eosinophilic cytoplasm and hyperchromatic nucleus (hematoxylin and eosin stain, × 400 magnification)

**Fig. 2:**
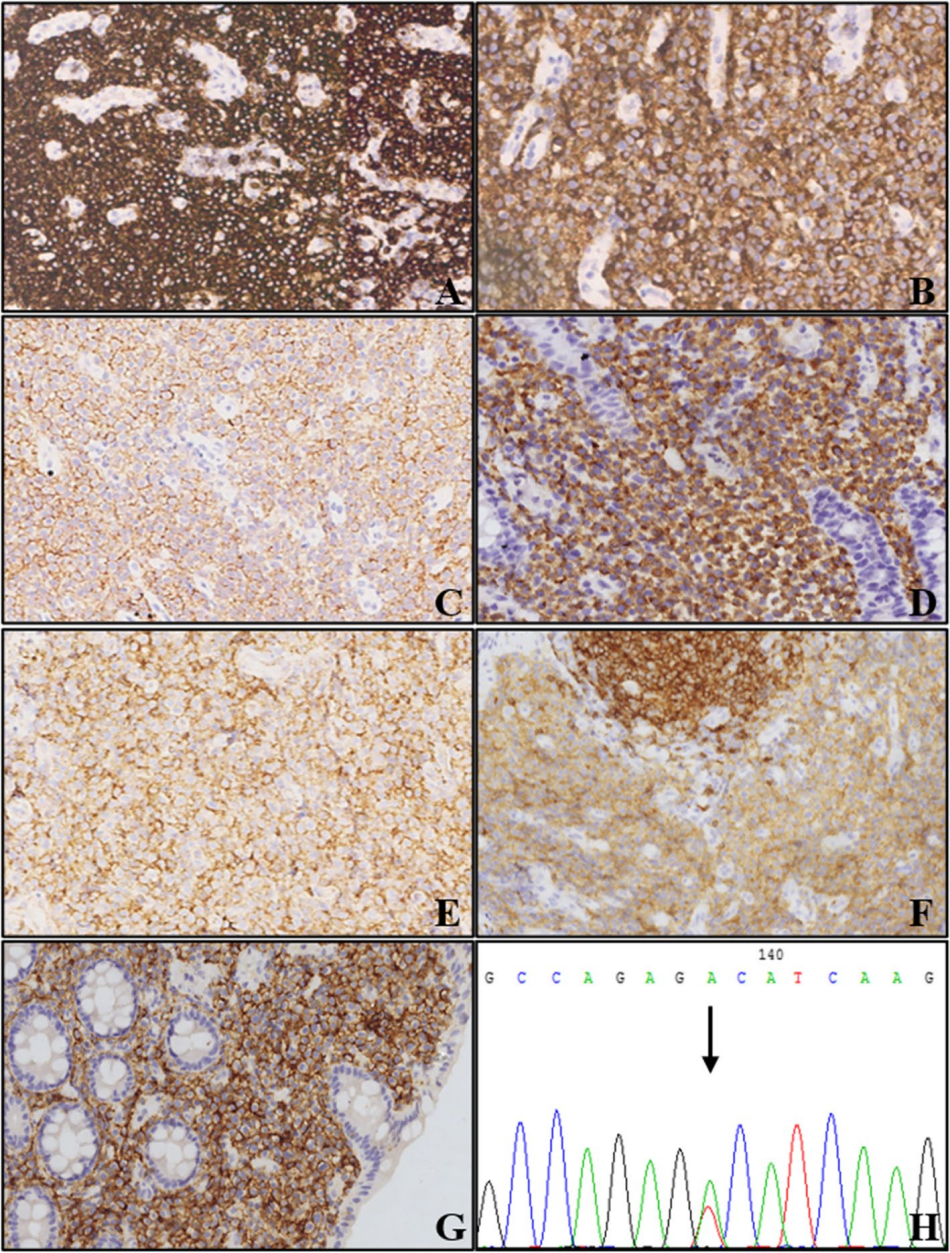
Immunohistochemical and molecular pathological findings of the patient with systemic mastocytosis. Diffuse expression of CD45 (**A**), CD117 (**B**), CD25 (**C**), CD68 (**D**), CD123 (**E**), CD4 (**F**), and CD56 (**G**) in mast cells (immunohistochemistry, × 200 magnification). **H** The missense mutation (c.2447A > T, p.D816V) in the exon 17 of KIT gene

**Fig. 3:**
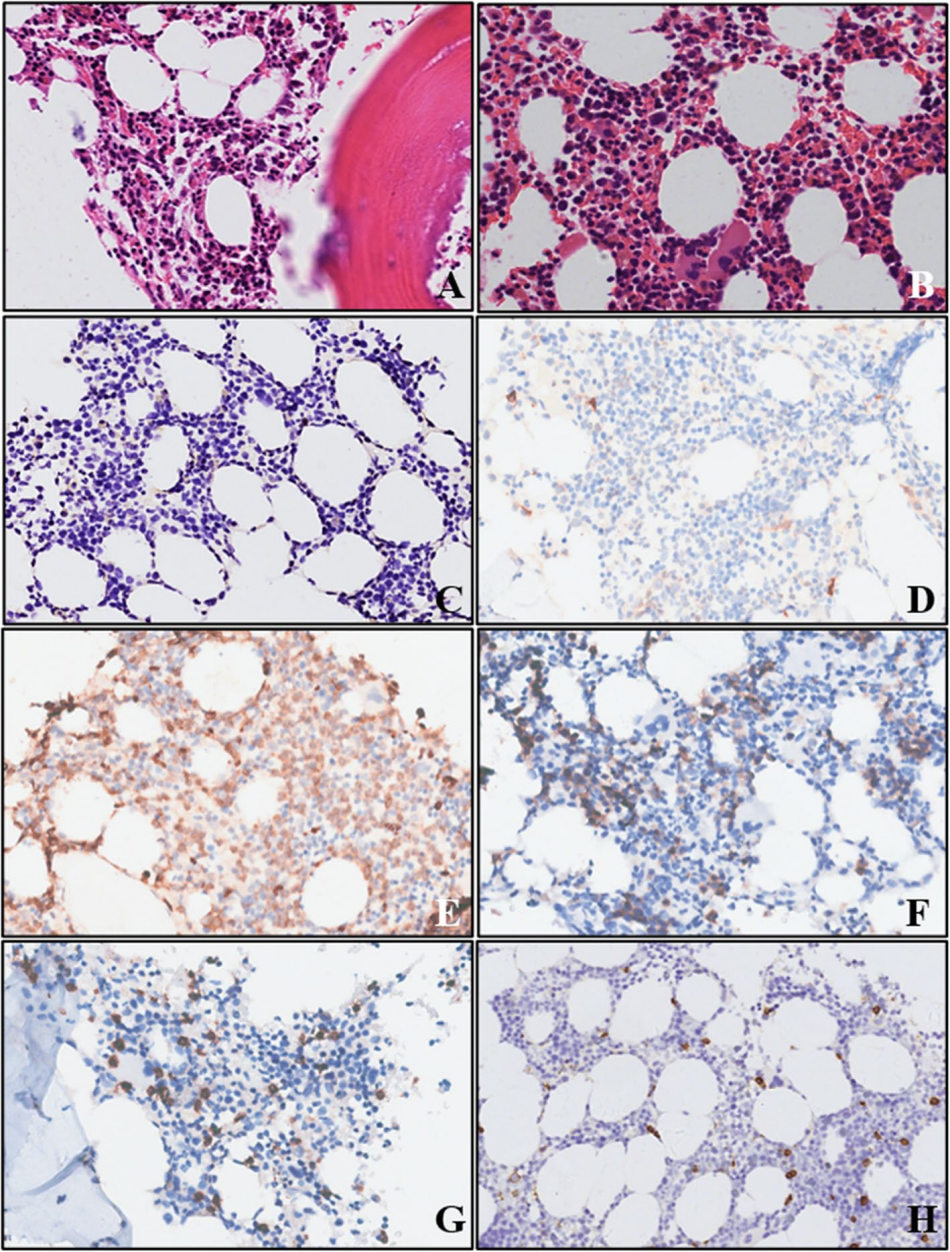
Bone marrow biopsy. **A** and **B** Normocellular marrow with trilineage hematopoiesis (hematoxylin and eosin stain, **A**, × 200 magnification; **B**, × 400 magnification). **C** and **D** No obvious aggregation of mast cells, as shown by CD25 (**C**) and CD117 (**D**) immunostaining (immunohistochemistry, × 200 magnification). **E**, **F**, **G**, **H** Expression of MPO (**E**), CD71 (**F**), CD3 (**G**), and CD20 (**H**) as the erythroid, myeloid, and lymphoid markers, respectively (immunohistochemistry, × 200 magnification)

The colonoscopy examination showed a 3.0 cm × 3.0 cm protuberant lesion in the cecum with erosion, necrosis, and ulcer formation on the surface (Fig. 1A). The tissue was tough and prone to bleeding during the biopsy. Histologically, the lamina propria was markedly expanded by medium-sized round/oval cells with abundant eosinophilic cytoplasm and hyperchromatic nucleus (Fig. 1B and C). The cells were slightly atypical and admixed with numerous eosinophils (Fig. 1C and D). Immunohistochemical analysis showed that the round/oval cells were negative for the epithelial markers, such as CKpan, cam5.2, EMA, CK7, and most lymphohematopoietic markers, such as CD20, CD79ɑ, CD3, CD5, CD8, CD21, CD23, BCL2, BCL6, CD10, mum-1, cyclin-D1, TDT, GranB, TIA-1, perforin, MPO, CD138, CD38, as well as other markers, such as CD163, langerin, CD1a, S-100, CD34, and EBER, but were diffusely positive for CD45 (Fig. 2A), CD117 (Fig. 2B), CD25 (Fig. 2C), CD68 (Fig. 2D), CD123 (Fig. 2E), CD4 (Fig. 2F), CD56 (Fig. 2G), and CD35. The staining intensity of CD4 was slightly lesser than that of small lymphocytes. The Ki-67 proliferation index in the disease area was 50%. Giemsa staining showed purplish-red granules in a small number of cells. Molecular analysis revealed missense mutation (D816V) in the exon 17 of KIT gene by Sanger sequencing (Fig. 2H), without clonal immunoglobulin (Ig)/T-cell receptor (TCR) rearrangement and BCR-ABL fusion. No chromosome aberration was observed by karyotyping analysis. Further bone marrow biopsy showed normocellular marrow with trilineage hematopoiesis and no mast cell aggregation (Fig.3). No primitive cells were found in bone marrow smear and peripheral blood smear (not shown).

**Fig. 4:**
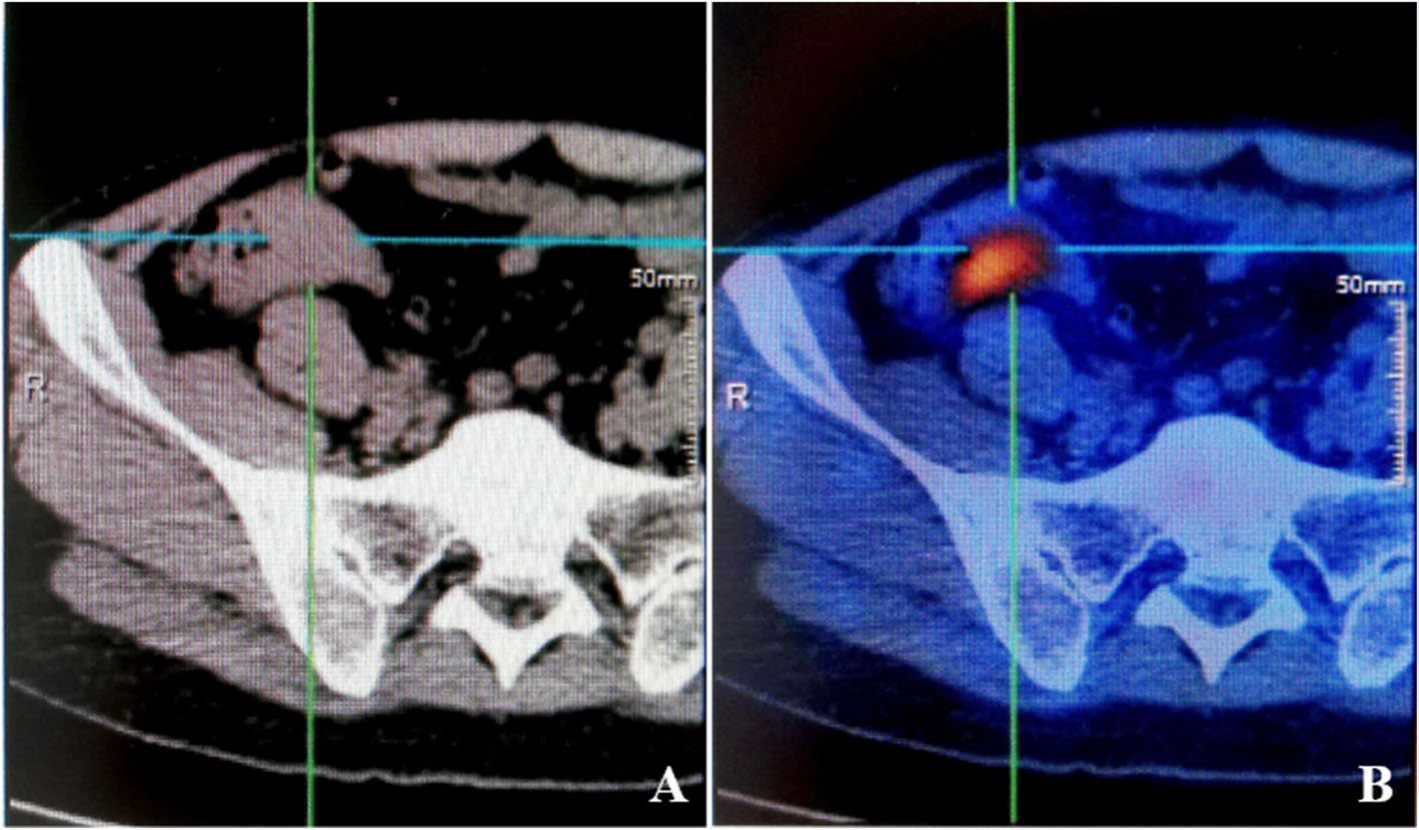
PET/CT findings. **A** Focal thickening of the cecum wall, about 13 mm. **B** Increased FDG uptake of the lesion

Laboratory examinations identified increased serum tryptase level (38.56 ng/ml, normal range 0–8 ng/ml). PET/CT showed local cecum wall thickening with increased FDG uptake (Fig. 4) and mild enlargement of adjacent lymph nodes. Vital signs and physical examination showed no obvious abnormalities. The patient’s body weight decreased by about 2.5 kg in recent 5 months.

A final diagnosis of KIT D816V-mutant aggressive SM involving intestinal tract was established based on the combination of morphologic, immunohistochemical, molecular, and clinical findings.

The patient was given avapritinib at an initial dosage of 200 mg once daily. After a month of treatment, the symptoms of diarrhea and abdomen discomfort ceased and the level of serum tryptase returned to normal ranges. Meanwhile, the patient’s memory was impaired, which recovered by gradually adjusting the drug dosage to 75 mg once daily. The patient underwent a routine ultrasound examination once a month by one designated doctor. Ultrasound results showed that the intestinal lesions and adjacent lymphadenopathy subsided after 1 month of treatment. The patient was followed up for 12 months and no recurrence was observed at the adjusted avapritinib dose (75 mg once daily).

## Discussion and conclusions

Mast cells are derived from hematopoietic stem cell precursors and play an important role in the genesis and manifestations of allergic response [5]. Mast cells are also associated with a variety of diseases, such as malignant tumors, Crohn’s disease, arthritis, coronary artery disease, and osteoporosis [5]. Normal mast cells are loosely distributed with abundant cytoplasm, round or oval nuclei, low nuclear/cytoplasmic ratio, and inconspicuous nucleoli. In contrast, neoplastic mast cells usually cluster together and display atypical or spindle cytological morphology with no or few mitotic figures. Immunohistochemically, although mast cells are bone marrow-derived, they lack expression for granulocyte and monocyte lineage antigen, and even most T and B cell antigens, but express CD45, CD117, tryptase, and CD68. Neoplastic mast cells further express CD2 and CD25, which are considered specific for mast cell clonality and could be used to confirm the diagnosis of mastocytosis [6–8].

SM, a subtype of mastocytosis, is a diagnosis made by integrating clinical, laboratory, morphological, immunohistochemical, and molecular genetic data. According to the WHO classification of tumors of hematopoietic and lymphoid tissues [1], the diagnosis of SM needs to fulfill the following either 1 major criterion and 1 minor criterion, or 3 minor criteria. (1) Major criterion: multifocal compact infiltrates of mast cells (≥15) in extracutaneous organ(s); (2) Minor criteria: A. Presence of >25% atypical or spindle-shaped mast cells in extracutaneous organ(s); B. Detection of KIT D816V mutation in extracutaneous organ(s); C. Expression of CD25, with or without CD2, in extracutaneous mast cells; D. Serum tryptase concentration >20 ng/mL (excluding cases with associated clonal myeloid neoplasm). Based on the severity and symptoms, SM is further subdivided into five phenotypes, namely indolent/smoldering SM and advanced SM including SM with an associated hematological neoplasm, aggressive SM, and mast cell leukemia. In our case, diffuse infiltrative oval and atypical mast cells were found in the lamina propria of intestinal mucosa; these cells were CD25 positive and contained D816V mutation in the exon 17 of KIT gene; serum tryptase elevated (38.56 ng/ml), which met 1 major criterion and 4 minor criteria. Combined with the symptoms such as abdomen discomfort, diarrhea, weight loss, and local lymphadenopathy, as well as no primitive cells in bone marrow smear and peripheral blood smear, intestinal aggressive SM was diagnosed.

Up to 80% of patients with SM are found to have gastrointestinal symptoms if a thorough medical history is taken [9]. The gastrointestinal symptoms are usually caused by the downstream effect of mediators released by mast cells on gastrointestinal tissues, and occasionally by the direct infiltration of neoplastic mast cells in the gastrointestinal tract [10]. The most common gastrointestinal symptom is diarrhea, with an incidence of 43% [11]. Besides, SM tends to involve bone marrow (90% of SM) [9], which usually presents as the aggregation of mast cells in perivascular and/or paratrabecular bone marrow regions. However, in our case, the evidence of bone marrow involvement by SM is insufficient as neither obvious mast cell aggregation nor aberrant CD25 expression was detected.

Morphologically, there exists diversity in the intestine involvement of SM [12, 13]. In advanced cases, mast cells densely infiltrate the mucosal layer, resulting in an obvious reduction of mucosa glands. However, in many early cases, clonal mast cells only slightly proliferate, and mucosal biopsy shows scattered or a small amount of mast cell infiltration. Coupled with the morphological similarity between mast cells and histiocytes, it is easy to be mistaken for histiocyte proliferation and missed diagnosis. Clues in the identification of scattered neoplastic mast cells include the slightly expanded spacing between lamina propria glands, the accumulation of lightly stained cells with relatively consistent morphology, and the infiltration of eosinophils [14].

The diagnosis of SM is difficult due to its rarity and atypical clinical presentation. The similarity of the clinical and histopathological features between BPDCN and our case makes the diagnosis further challenging. Clinically, both diseases are rare hematologic malignancies with heterogeneous clinical presentation and have a high frequency of cutaneous and bone marrow involvement, as well as lymph node and visceral organ involvement [1]. Microscopically, the cells of both neoplasms appear diffuse, monomorphous, and medium-sized. Most similarly, both neoplastic cells are negative for the lineage-associated antigens and the clonal Ig/TCR rearrangement, although TCR rearrangement has been occasionally reported in patients with BPDCN [1]. The immunoreactivity for CD45, CD117, CD68, CD123, CD4, and CD56 that is observed in our case can also be detected in BPDCN, leading to potential diagnostic pitfall. CD123, CD4, and CD56 are important markers for BPDCN diagnosis [1]. It is not surprising that SM expresses CD123 [15]. However, SM with a diffuse expression of CD4 and/or CD56 has rarely been reported [7, 16, 17], which brings hesitation to our diagnosis to a certain extent. The staining intensity of CD4 in our case was slightly lower than that of small lymphocytes. This is consistent with Soilleux et al. [16], who demonstrated the first case of mastocytosis with CD4 expression and observed a similar CD4 staining pattern in 5 other cases. The coexistence of aberrant CD25 expression and KIT gene alteration in our case provides a pivotal distinguishing point between the two diseases.

A majority of adult SM patients carry somatic gain-of-function mutations of KIT, most are at amino acid 816 in exon 17, where aspartic acid is substituted by valine (D816V) (>90%) [18]. Other rare KIT mutations include V560G, D815K, D816Y, insVI815–816, D816F, D816H, D820G, and F522C [19]. KIT encodes a receptor tyrosine kinase (CD117 or stem cell factor receptor), which plays an important role in mast cell growth, development, migration, and function by binding to cytokine ligand (stem cell factor) [20]. KIT D816V mutation can constitutively activate tyrosine kinase, and served as a driver mutation for SM [21]. However, KIT D816V mutation is not specific to SM, it can also be observed in acute myeloid leukemia and melanoma [20].

SM is considered incurable, and patients with aggressive SM have a worse survival rate due to organ damage, with an overall median survival of 41 months [19]. The treatment of SM is highly correlated with KIT gene status. For a few SM patients with non-D816V KIT mutations, significant clinical responses have been observed with imatinib treatment [22]. Imatinib binds to the intracellular pocket located within tyrosine kinases, thereby blocking ATP and peptide substrate binding and preventing phosphorylation, secondary growth receptor activation, and downstream signaling [23, 24]. However, for most patients with KIT D816V mutations, the therapeutic effect of imatinib is limited, as the KIT tyrosine kinase ligand-independent activation caused by D816V mutation may induce primary resistance against imatinib [22]. Recently, a second-generation tyrosine kinase inhibitor agent, avapritinib, has been developed. Avapritinib is a pyrrolotriazine derivative that is particularly against KIT and PDGFRA activation loop mutants including D816V. In clinical trials, the application of avapritinib in SM has achieved satisfactory clinical and histological results and has been recently approved by the U.S. Food and Drug Administration for the treatment of advanced SM with a recommended dosage of 200 mg orally once daily [19, 25]. The most common side effects of avapritinib include periorbital edema, diarrhea, nausea, and asthenia, occurring in 20% or more patients [25]. As the first case report using avapritinib for aggressive SM in China, our patient had complete remission of symptoms and serum tryptase level returned to normal level within 1 month of treatment. The intestinal lesions and local lymphadenopathy also became subsided. However, the patient experienced a rare side effect, memory impairment, which was gradually recovered by reducing the drug dosage from 200 mg to 75 mg per day. At this point, the benefits of avapritinib outweighed the side effects. The patient has remained on stable disease at the dosage of 75 mg avapritinib once daily.

In conclusion, SM is very rare, and therefore, confirmation of a diagnosis can pose a significant challenge. The pathologist and clinician must have a high degree of suspicion, particularly in patients presenting with long-term diarrhea symptoms and hematopoietic/lymphoid-appearing tumors. SM with CD123, CD4 and CD56 co-expression has rarely been reported. The rare side-effect of memory impairment in this case helps to accumulate the experience of avapritinib in treating KIT D816V-mutant SM.

## Data Availability

The datasets used and/or analyzed during the current study are available from the corresponding author on reasonable request.

## Abbreviations

SM: Systemic mastocytosis
WHO: World Health Organization
BPDCN: Blastic plasmacytoid dendritic cell neoplasm
Ig: Immunoglobulin
TCR: T-cell receptor
